# Barbatic Acid Offers a New Possibility for Control of *Biomphalaria Glabrata* and Schistosomiasis

**DOI:** 10.3390/molecules22040568

**Published:** 2017-03-31

**Authors:** Mônica Cristina Barroso Martins, Monique Costa Silva, Hianna Arely Milca Fagundes Silva, Luanna Ribeiro Santos Silva, Mônica Camelo Pessoa de Azevedo Albuquerque, André Lima Aires, Emerson Peter da Silva Falcão, Eugênia C. Pereira, Ana Maria Mendonça Albuquerque de Melo, Nicácio Henrique da Silva

**Affiliations:** 1 Departamento de Bioquímica e Fisiologia, Universidade Federal de Pernambuco, Recife, PE 50670-901, Brazil; monicabarmartins@hotmail.com (M.C.B.M.); nickcosta.91@gmail.com (M.C.S.); hiannaamfs@gmail.com (H.A.M.F.S.); nhsilva@uol.com.br (N.H.d.S.); 2 Departamento de Radiobiologia, Universidade Federal de Pernambuco, Recife, PE 50670-901, Brazil; luannaribeiro_lua@hotmail.com (L.R.S.S.); amdemelo@hotmail.com (A.M.M.A.d.M.); 3 Laboratório de Imunopatologia Keizo Asami LIKA, Universidade Federal de Pernambuco, Recife, PE 50670-901, Brazil; jcmonica@globo.com (M.C.P.d.A.A.); andrelima26@gmail.com (A.L.A.); 4 Laboratório de Síntese e Isolamento Molecular, Centro Acadêmico de Vitória de Santo Antão, Universidade Federal de Pernambuco, Vitória de Santo Antão, PE 50670-901, Brazil; emerson_falco@yahoo.com.br; 5 Departamento de Ciências Geográficas, Centro de Filosofia e Ciências Humanas, Universidade Federal de Pernambuco, Recife, PE 50670-901, Brazil

**Keywords:** *Cladia aggregata*, embryotoxicity, molluscicidal activity, environmental toxicity, lichen substances, mollusks

## Abstract

This study evaluated the biological activity of an ether extract and barbatic acid (BAR) from *Cladia aggregata* on embryos and adult mollusks of *Biomphalaria glabrata*, cercariae of *Schistosoma mansoni* and the microcrustacean *Artemia salina*. The ether extract and BAR were obtained by successive extractions with diethyl ether. The obtained extracts were analyzed using thin-layer chromatography (TLC), high-performance liquid chromatography (HPLC), proton nuclear magnetic resonance (^1^H-NMR) and infrared (IR) spectroscopy. The results demonstrated that the ether extract exerted embryotoxic effects at 50 and 100 µg/mL and molluscicidal effects at 20 and 25 µg/mL. BAR exhibited no embryotoxicity, and its molluscicidal concentration was equal to that of the ether extract. However, after 60 min of exposure, 1 µg/mL BAR presented cercaricidal activity against the parasite *S. mansoni* at the second larval stage. Neither substance induced toxicity against *A. salina*. These results indicate the potential molluscicidal activities of the ether extract and BAR against *B. glabrata* and *S. mansoni* cercariae. In addition to these effects, there was a lack of toxicity against the aquatic environment and no damage to the biota, indicating the potential of these products for large-scale control and/or eradication of schistosomiasis.

## 1. Introduction

Human schistosomiasis, a parasitic disease caused by trematode worms of the *Schistosoma* genus, is one of the most prevalent and debilitating parasitoses among neglected tropical diseases. It has been estimated that approximately 261 million people require treatment for schistosomiasis in 78 countries in Africa, Asia and South America [1,2].

In Brazil, the snail *B. glabrata* is the most important vector for schistosomiasis and is associated with high rates of disease infection and transmission. This species can be found in aquatic environments, where the snails spawn and release cercariae of *S. mansoni* (infective larval stage) [1,3], which are generally eradicated with synthetic chemicals, such as niclosamide, as recommended by the World Health Organization (WHO) [4]. However, although niclosamide at low concentrations efficiently eliminates snails and cercariae at all phases of the life cycle, it is both light-sensitive and toxic to fish, amphibians and aquatic plants [5]. In addition, the costs associated with the application of niclosamide are high, and the snails can develop resistance to this synthetic molluscicide [6]. Thus, natural products from plants and/or other organisms [7,8] could be considered promising sources of novel substances with molluscicidal activity.

In nature, organisms utilize various nutritional strategies, including lichenization, which is a symbiotic association between a fungus (usually an ascomycetes), an algae (often of the Chlorophyceae class), and/or a cyanobacterium. This arrangement ensures that the lichen can benefit from secondary metabolites, which have extensive biological uses [9]. Because of their different biological properties, including antimicrobial [10,11], anti-tumor [12,13,14,15], antiherbivore [16], insecticidal [17], and molluscicidal activities [18], secondary metabolites, such as polyphenolic compounds from lichens, have been used in folk medicine since ancient times [19].

Asplund et al. [20] reported that secondary metabolites from thalli lichen have an important ecological function in preventing slugs and land snails from feeding on these species. According to Lawrey [21], the food preference of some land mollusk species for thalli free of depsides and dibenzofurans (e.g., stictic and usnic acids, respectively) might be related to palatability, indirect effects on intestinal microbiota, antibiosis, and the direct toxicity of these substances to mollusks.

For this reason, it was hypothesized that barbatic acid (BAR) might also exert effects on aquatic mollusks, such as adult *B. glabrata* or larval *S. mansoni*. BAR is an important depside that acts on the photosynthesis photosystem II [22], inhibits non-redox reactions during the synthesis of leukotrienes (LTB4), suppresses keratinocyte proliferation [23], and demonstrates bactericidal activity against *Staphylococcus aureus* [11] as well as antitumor activity [24,25]. However, there are no reports of any molluscicidal activity of this metabolite.

In this context, phenolic compounds from lichens are a promising source for biomolecules. Certain polyphenolic compounds present in higher plants have been shown to exhibit molluscicidal properties against different species of mollusks [26,27]. Therefore, given their similar metabolic origins, we hypothesized that both the ether extract of and BAR from *C. aggregata* (lichen) efficiently act on embryos and adults of *B. glabrata* and *S. mansoni* cercariae. In addition to testing this hypothesis, we further investigated whether these molecules exert toxic effects on the environment through bioassays using brine shrimp (*Artemia salina*) as a bioindicator.

## 2. Results

### 2.1. Chemical Analysis

**Figure 1:**
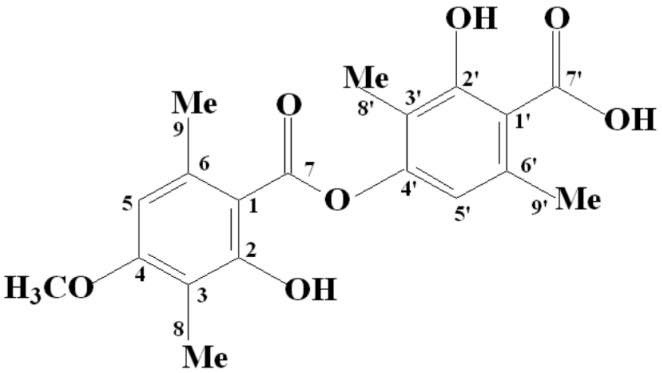
Structure of BAR.

Lichens possess several characteristic phenolic chemotypes whose occurrence can vary depending on season and collection site. This phenotypic plasticity is a typical feature of *C. aggregata* and depends primarily on environmental conditions. For this reason, different chemotypes, such as stictic, constictic, norstictic and cryptostictic acids, can be found. In contrast, in Brazil, BAR is the dominant substance found in the species, which is consistent with the findings obtained in this study. Chromatographic analysis of BAR (C_19_H_20_O_7_) from *C. aggregata* revealed a *R*_f_ value of 0.43 for TLC, whereas HPLC revealed a R_T_ value of 19.74 min and a purity of 96.6%. The ether extract contained both BAR (RT 19.7 min) and STI (2.8 min) at respective concentrations of 93% and 0.2%, as well as other minor compounds that remain unidentified. ^1^H-NMR and ^13^C-NMR data confirmed that the molecular structure of BAR was in accordance with the one reported by Martins et al. [11] (Figure 1).

### 2.2. Toxicity of the Ether Extract of And BAR from C. aggregata on Embryo and Adult Mollusks

**Figure 2:**
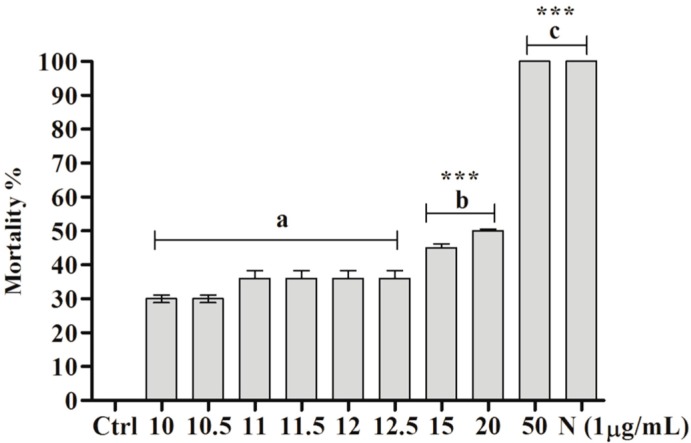
Effect of ether extract (µg/mL) of *C. aggregata* on *B. glabrata* embryos: Ctrl—negative control (0.5% ethanol + filtered and dechlorinated water); N—niclosamide (1 µg/mL). The asterisks (***) indicate significant differences (*p* < 0.0001) compared with the negative control. The letter “**a**” indicates that the treatments presented no statistically significant differences between groups, the letter “**b**” indicates significant differences compared with **a**, and the letter “**c**” indicates that the treatments (50 µg/mL and N) did not present significant differences between groups.

Significant embryotoxic activity was demonstrated for the ether extract of *C. aggregata* (*** *p* < 0.0001, F = 380.3). Statistically significant differences were obtained at concentrations higher than 10 µg/mL (*** *p* < 0.0001) compared with the negative control (water and 0.5% ethanol). Furthermore, 100% mortality was observed at 50 µg/mL (Figure 2). No significant difference was detected between the concentrations of 10, 10.5, 11, 11.5, 12, and 12.5 µg/mL and the negative control or between 50 µg/mL (95% CI of diff: −7.729 to 7.729) and the positive control. However, a significant difference was found between 15 and 50 µg/mL (95% CI of diff, −62.73 to −47.27, *** *p* < 0.0001) and between 20 and 50 µg/mL (95% CI of diff, −57.73 to −42.27, *** *p* < 0.0001). The LC_50_ of the ether extract on embryos was 19.9 µg/mL, whereas BAR exerted no toxic activity.

**Figure 3:**
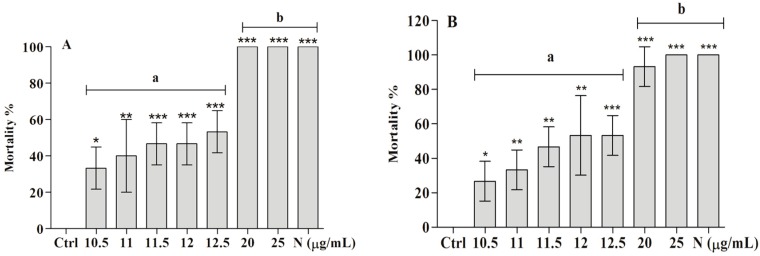
Molluscicidal activity (µg/mL) of the ether extract (**A**) and BAR (**B**) against *B. glabrata* snails. Ctrl—negative control (0.5% ethanol + filtered and dechlorinated water); N—niclosamide (1 µg/mL). The significance levels of the differences compared with the negative control (ANOVA) are indicated with * (*p* < 0.05), ** (*p* < 0.01) and *** (*p* < 0.0001). The letters “**a**” and “**b**” indicate that the groups did not and did present significant differences (Tukey’s test, *** *p* < 0.0001), respectively.

Treatment with the ether extract revealed significant differences for concentrations higher than 10.5 µg/ mL (ANOVA, *p* < 0.0001, F = 35.68) compared with the control (Ctrl). At 20 µg/mL, there was 100% mortality (*** *p* < 0.0001) compared with the Ctrl, demonstrating the efficiency of the ether extract at lower concentrations. However, Tukey’s test showed no significant difference among concentrations of 11, 11.5, 12 and 12.5 µg/mL. Among the treated groups, statistically significant differences were found between 10.5 and 20 µg/mL (95% CI of diff, −4.790 to −1.8, *** *p* < 0.0001), 11 and 20 µg/mL (95% CI of diff, −4.457 to – 1.5, *** *p* < 0.0001), 11.5 and 20 µg/mL (95% CI of diff, −4.124 to −1.2, *** *p* < 0.0001), 12 and 20 µg/mL (95% CI of diff, −4.124 to −1.2, *** *p* < 0.0001) and 12.5 and 20 µg/mL (95% CI of diff, −3.790 to −0.8, *** *p* < 0.0001). Furthermore, no statistically significant difference was detected between 20 or 25 µg/mL and the positive control (niclosamide), proving the efficiency of the ether extract (Figure 3A). The LC_50_ value for the extract was calculated as 11.9 µg/mL.

Similar to the ether extract, BAR showed substantial molluscicidal activity against snails (ANOVA, *** *p* < 0.0001, F = 27.78) at a concentration as low as 11 µg/mL (* *p* < 0.05) compared with the negative control. However, the greatest molluscicidal activity was observed at 25 µg/mL, which showed 100% lethality. The LC_50_ value for BAR was 11.9 µg/mL. Among the treated groups, no significant difference was detected between the concentrations of 10.5, 11, 11.5, 12 and 12.5 µg/mL (Figure 3B). Significant differences were found between 10.5 and 20 µg/mL (95% CI of diff, −99.71 to −33.63, *** *p* < 0.0001), 10.5 and 25 µg/mL (95% CI of diff, −106.4 to −40.29, *** *p* < 0.0001), 11 and 20 µg/mL (95% CI of diff, −93.04 to −26.96, *** *p* < 0.0001), 11 and 25 µg/mL (95% CI of diff, −99.71 to −33.63, *** *p* < 0.0001), 11.5 and 20 µg/mL (95% CI of diff, −79.71 to −13.63, ** *p* < 0.01), 11.5 and 25 µg/mL (95% CI of diff, −86.37 to −20.29, *** *p* < 0.0001), 12 and 20 µg/mL (95% CI of diff, −73.04 to −6.960, * *p* < 0.05), 12 and 25 µg/mL (95% CI of diff, −79.71 to −13.63, ** *p* < 0.01), 12.5 and 20 µg/mL (95% CI of diff, −73.04 to −6.960, * *p* < 0.05), and 12.5 and 25 µg/mL (95% CI of diff, −79.71 to −13.63, ** *p* < 0.01). There was no significant difference between 20 and 25 µg/mL and the positive control, proving the efficiency of both concentrations compared with niclosamide.

Mollusk spawns were treated with the ether extract and BAR, and those that survived were collected and analyzed until the first generation hatched (F1). Interestingly, although 66% of the mollusks treated with 10.5 µg/mL ether extract survived, they were not able to spawn. The same result was observed following exposure to 11, 11.5, 12.5 and 20 µg/mL BAR, clearly demonstrating that these substances interfered with the mollusk spawning process (Table 1).

### 2.3. Toxicity of BAR from C. aggregata to S. mansoni Cercariae

Table 2 shows the partial lethality of BAR on *S. mansoni* cercariae in relation to exposure time. Cercaricidal activity was first detected after exposure to a concentration of 0.25 µg/mL for 30 min (+), and more than 50% lethality (++) was obtained after exposure to a concentration of 0.5 µg/mL for 60 min. Complete elimination of cercariae (+++) was observed after exposure to 1 µg/mL for 60 min. The time required for complete elimination of cercariae decreased with increasing concentration, with 30 min being needed at a concentration of 10 µg/mL and 15 min at a concentration of 100 µg/mL.

**Figure 4:**
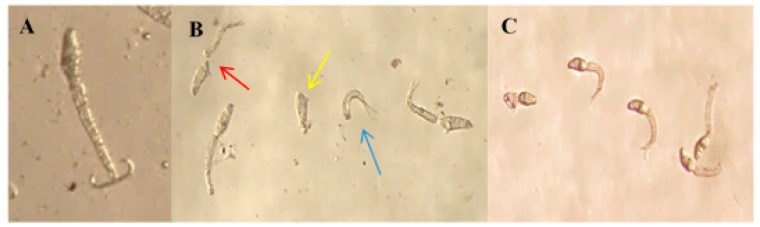
Cercariae of *S. mansoni* exposed to BAR. (**A**) Image of cercariae treated with 0.5% ethanol and filtered water, showing preservation of the body and tail; (**B**) Image of cercariae exposed for 30 min to 1 µg/mL BAR, showing a split between the body and tail (red arrow); an individual body (yellow arrow) and tail (blue arrow) are also displayed; (**C**) Image of dead cercariae after exposure to niclosamide (1 µg/mL). 40× magnification.

During the treatment, there were changes in engine behavior, such as atypical rotation and vibrations. Specifically, the cercariae exhibited slow rotation around their own axes, creeping and different intensities of contractions that increased as the concentration of BAR was increased. These findings highlight the dose-dependent effect of the substance. Figure 4 shows images highlighting the significant differences among the treatments: in the negative control group (A), the cercariae presented normal rotation and vibration motility accompanied by preservation of the body and tail, whereas the treatment group (B) showed separation of the body and cercariae tail, and the positive control group (C) resulted in cercariae death. However, niclosamide did not cause separation of body and tail, as was observed in the group treated with BAR.

**Figure 5:**
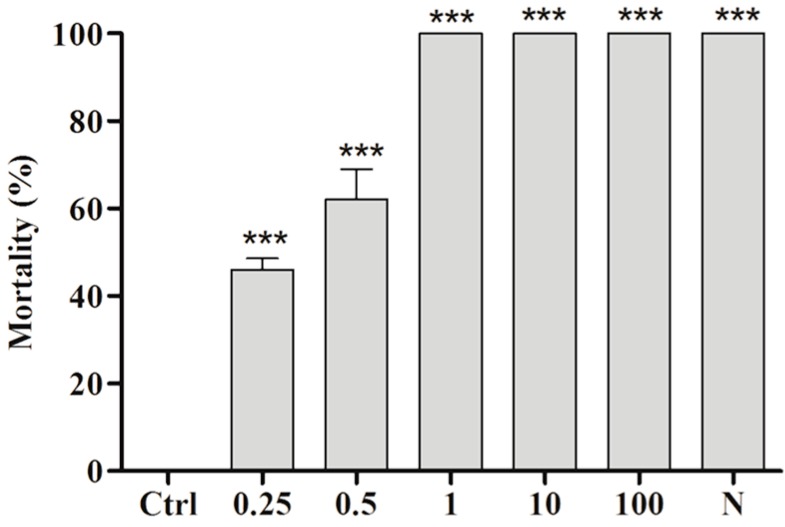
Cercaricidal activity of BAR against *S. mansoni* at the end of the 120-min exposure period. Ctrl (0.5% ethanol and filtered water); N (niclosamide at 1 µg/mL). The results were compared with the Ctrl; *** *p* < 0.001.

Figure 5 displays the lethality of BAR at the end of the experiment (120 min), at which time all concentrations showed significant differences compared with the Ctrl. The average lethality values obtained for 0.25, 0.5, 1, 10 and 100 µg/mL were 46 ± 4.58 (*p* < 0.0001), 62 ± 12.0 (*p* < 0.0001), 100 (*p* < 0.0001), 100 (*p* < 0.0001), and 100 (*p* < 0.0001), respectively. The LC_50_ was calculated as 0.45 µg/mL.

### 2.4. Toxicity of the Ether Extract of and BAR from C. aggregata against A. salina

**Figure 6:**
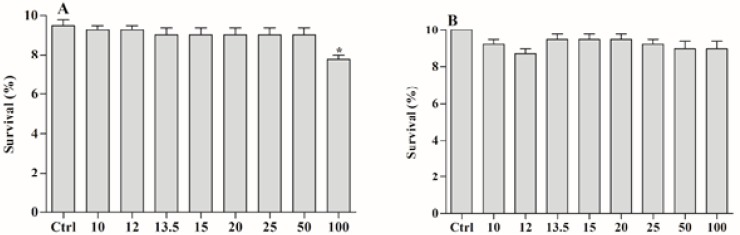
Toxicity of ether extract (**A**) and BAR (**B**) on *Artemia salina*. The ctrl was 0.5% ethanol and seawater. * *p* < 0.05.

Ecotoxicity assays are important for establishing safe environmental parameters regarding the use of xenobiotics. For this reason, we tested both the ether extract and BAR on the environmental bioindicator *A. salina*. The results showed that both products from *C. aggregata* were nontoxic against *A. salina* at all tested concentrations, with *p* = 0.0904 and F = 1.967 for the extract (Figure 6A) and *p* = 0.1710 and F = 1.601 for BAR (Figure 6B). However, at 100 µg/mL, the effects of the extract were significant (*p* < 0.05, 95% CI of diff, 3.418 to −0.08152).

## 3. Discussion

Research correlating lichen substances and schistosomiasis vectors is still novel because few reports on these topics have been published. In this context, Martins et al. [18] evaluated the molluscicidal activity of potassium usnate in a pioneering study of this type of biological activity and demonstrated that *B. glabrata* shows embryotoxicity and molluscicidal activity at 1 µg/mL and 10 µg/mL, respectively. However, contrary to potassium usnate, the BAR tested in this study did not exert any effect on embryos. In contrast, the molluscicidal activity of BAR was significant (20 µg/mL) and in accordance with the standards recommended by the WHO [4] because it eliminated 90% of the same mollusk population, like potassium usnate. Even though potassium usnate is a lichen phenolic modified to a salt form, its solubility in water is higher than that of BAR, a property that could enhance its ability to induce mortality compared with that of BAR. Because potassium usnate is a salt that can be structurally modified from dibenzofurane derivative, a lichen phenolic (usnic acid). The findings obtained for the organic extract were significant, showing that it could serve as a molluscicide and that it exhibits embryotoxic activity at low concentrations (as low as 10 µg/mL), even though it does not cause 100% mortality. According to HPLC analysis, the BAR content in the extract is over 90%; thus, we expected to obtain a higher LC_50_ for BAR than for the extract. Additionally, we analyzed the chemistry of the ether extract, which contains other substances capable of potentializing its biological effects on mollusks. Importantly, the concentrations of lichen substances used in this study are lower than those found in some plant extracts, showing the efficiency of BAR on mollusks. For example, a chloroform extract of *Solanum siniacum* displayed molluscicidal activity at 64.4 µg/mL [28].

Ecologically, lichen substances play a key role in thallus maintenance and act as protectors of small, mobile herbivores (insects, snails and mollusks) [20]. Lawrey [21] reported that snails of the species *Pallifera varia* (Hubricht) avoid feeding on lichen species such as *Xanthoparmelia cumberland* (Gyelnik) Hale, which contain usnic, norstictic and stictic acids, and *Huilia albocaerulescens* (Wulfen) Hertel, which produce constictic and stictic acids. The food preferences of invertebrates were reported by Fröberg et al. [29] and Benesperi and Tretiach [30], who disclosed that snails preferentially feed on different parts of lichens. Gauslaa [31] described the food preferences of lichens that do not have secondary metabolites, even though metabolites can be extracted using the acetone rinsing method, which indicates these substances cause some type of toxicity to these animals. Lawrey [21] believes that in addition to reducing the palatability of lichens, the substances show direct toxicity or indirect antibiotic effects on the gut microbiota of predator organisms. However, we believe that this effect cannot be extended to our findings because neither the ether extract nor BAR was toxic to *A. salina*, an environmental bioindicator species, and these preliminary results of environmental toxicity with *A. salina* indicate that BAR could be non-toxic or less toxic than niclosamide. Based on these findings, these substances are potentially safe for the environment.

Studies aiming to identify molluscicidal agents should consider methods to not only suppress parasite vectors but also combat the infectious stage of *S. mansoni*. A substituted pyridine pentahydrate (2,6-dimethyl-3,5-carboxydiethyl-4-phenylpyridine) was assayed against *B. glabrata* and *S. mansoni* cercariae and showed promising results, eliminating 90% of mollusks at 36.43 µg/mL and 100% of cercariae at 4 µg/mL within 30 min of exposure [32]. However, essential oil extracted from *Piper cubeba* L. was effective against cercariae of *S. mansoni* at 200 µg/mL [33], a concentration above that recommended for environmental applications according to the WHO [4]. Our results demonstrate the efficacy of BAR, which eliminated 100% of cercariae at a concentration of 1 μg/mL, the same concentration used for the niclosamide positive control.

A study of *Glinus lotoides* (Molluginaceae) showed that exposure of *S. mansoni* cercariae to an aqueous extract of the plant at a concentration of 18.7 µg/mL resulted in motility alterations that directly influenced the potential penetration of cercariae into mice and reduced the parasite load by 91.2% [34]. Similar motility alterations were observed in this the present study, suggesting that cercariae exposed to BAR at sub-lethal concentrations had reduced infectivity. Separation of body and tail has also been observed in other studies [35,36] and is likely a result of weaknesses in the tail structure. Although the mechanism of action has not yet been elucidated, it is believed that this process is associated with the actions of certain substances on a special connective structure between the body and tail [37].

Ravaglia et al. [38] indicated the importance of screening biologically active compounds for toxicity and verified the toxicity of extracts from *C. aggregata* on *A. salina*. Their results (LC_50_ = 690.6 μg·mL^−^^1^) differ from ours, which revealed that neither BAR nor the ether extract exhibited toxicity at lower doses, although the ether extract at a concentration of 100 μg/mL exhibited toxicity, potentially due to the combination of substances present in the extract. According to Ahti et al. [39], *C. aggregata* contains stictic, constictic, norstictic, and cryptostictic acids.

## 4. Materials and Methods

### 4.1. Extract Production and Purification of BAR from Cladia aggregata (Sw.) Nyl.

*C. aggregata* (50 g) was collected in Bonito, Pernambuco, Brazil at the coordinates 08°28′13″ S and 35°43′43″ W Gr. on 6 November 2010. A sample was deposited in the Herbarium UFP of the Universidade Federal de Pernambuco/UFPE, Brazil (Voucher No. 36431). BAR was obtained through successive extractions with a Soxhlet apparatus (30 °C) using diethyl ether. To isolate and purify BAR, the extract was successively washed with chloroform in a G4 funnel under pressure. BAR was obtained at high purity (>95%), as monitored through thin-layer and liquid chromatography. The molecular structure was determined by ^1^H-NMR and IR. Additionally details related to the extraction and purification of BAR and the preparation of organic extracts are provided by Martins et al. [11].

### 4.2. Mollusks

Pigmented mollusks of the species *B. glabrata* (Say, 1818) were obtained from São Lourenço da Mata, Pernambuco, and were reared in the Radiobiology Laboratory of the Department of Biophysics and Radiobiology of the Federal University of Pernambuco through successive generations. The mollusks were selected according to the diameters of their shells (10–14 mm) and were maintained in plastic tanks with filtered and dechlorinated water (pH 7.0, ±25 °C) and fed fresh lettuce (*Lactuca sativa*).

### 4.3. Embryotoxic Activity of the Ether Extract of and BAR from Cladia aggregata

To test embryotoxicity, we collected egg masses (*n* = 100) in the blastocyst phase and examined their viability using a stereomicroscope (Leica MZ6, Leica Microsystems, Wetzlar, Germany). The embryos were deposited on Petri dishes (6 mm) and treated with the ether extract from *C. aggregata* or BAR at concentrations of 1, 10, 10.5, 11, 11.5, 12, 12.5, 15, 20 and 50 µg/mL solubilized in ethanol (0.5%) at a final volume of 10 mL per plate for 24 h. The control groups were the following: 1 µg/mL niclosamide (N; positive control; Bayluscide, Bayer, Leverkusen, Germany), ethanol (0.5%; negative control) and filtered and dechlorinated water (Ctrl, negative control). The embryos were evaluated for malformation and/or mortality [40], and the experiment was performed in triplicate.

### 4.4. Molluscicidal Activity of the Ether Extract of and BAR from Cladia aggregata

To determine molluscicidal activity, a population of 400 mollusks was pre-selected and maintained in isolation for five days to confirm sexual maturity. Groups of mollusks (*n* = 5) were transferred to small aquariums (500 mL), where they were treated with either the ether extract or BAR (dissolved in 0.5% ethanol) from *C. aggregata* at 1, 10, 10.5, 11, 11.5, 12, 12.5, 20, 25, 50 and 100 µg/mL for 24 h. Two negative controls were used: filtered water and ethanol (0.5%) plus filtered and dechlorinated water (Ctrl). For the positive control, niclosamide (N) was used at 1 µg/mL. After a 24 h exposure period, the animals were washed in distilled water, left untreated, fed fresh lettuce (*L. sativa*) and observed for 96 h. The mollusks that survived the treatment were monitored, and the egg masses were again recorded and analyzed to evaluate the fertility and fecundity of the snails. Retraction of the mollusks into their shells and/or the release of hemolymph were used as indicators of death [41]. The experiment was performed in triplicate.

### 4.5. Cercaricidal Activity of the Ether Extract of and BAR from C. aggregata

Pigmented and infected *B. glabrata* mollusks were placed in a 200 mL beaker, submerged in distilled water and exposed to artificial light (60 W) for 2 h until the elimination of cercariae was achieved. To assay toxicity, approximately 100 cercariae suspended in distilled water were placed in a glass container and exposed to 2 mL of BAR solubilized in ethanol (0.5%) at 0.25, 0.5, 1.0, 10 and 100 µg/mL. The cercariae were observed using a stereomicroscope (Wild M3B, Heerbrugg, Switzerland) at intervals of 15, 30, 60 and 120 min after exposure and evaluated using the following parameters: complete elimination of cercariae (+++), elimination of more than 50% of cercariae (++), elimination of less than 50% of cercariae (+) and absence of lethality (−). Niclosamide at a concentration of 1 µg/mL and 0.5% ethanol plus filtered and dechlorinated water (Ctrl) were used as positive and negative control groups, respectively. The bioassay was performed in triplicate. The assaying of various time intervals allowed us to observe atypical rotations and vibrations of cercariae during the experiments, and the results were registered through images obtained with a digital camera coupled to a stereomicroscope (40×). After 120 min, the number of dead cercariae was counted, and the LC_50_ was calculated.

### 4.6. Environmental Toxicity Assays with Artemia salina

*A. salina* cysts (25 mg) were incubated in filtered seawater under artificial light at a temperature of 30 °C ether extract or BAR at concentrations of 1, 10, 12, 13.5, 15, 20, 25, 50 and 100 mg/mL solubilized in seawater and 0.5% ethanol. The tubes were incubated for 24 h, and survival was determined for each treatment. Two groups of negative controls were included: filtered seawater and 0.5% ethanol (Ctrl) plus filtered seawater. The experiment was performed in quadruplicate [42].

### 4.7. Statistical Analysis

Data were analyzed using GraphPad Prism 5.0 for Windows (GraphPad Software, San Diego, CA, USA). Significant differences were established through analysis of variance (ANOVA) and Tukey’s test at *p* < 0.05. The lethal concentrations (LC_50_) for the embryos, mollusks and *A. salina* were calculated via Probit analysis with 95% confidence intervals using StatPlus 168 2006 software (Soft Analyst, Vancouver, BC, Canada).

## 5. Conclusions

The presented results expand the available knowledge regarding the application of active metabolites for the control of schistosomiasis vectors. These findings should be of great interest to researchers in the fields of public health and environmental preservation because the studied molluscicidal substances showed great biological potential. In addition, their characteristics were in accordance with WHO standards, and they were nontoxic to the environment, as demonstrated through bioassays with *A. salina*.

From an environmental point of view, BAR appeared to be more efficient than the corresponding ether extract. Although BAR does not exert any effect on embryos, it is capable of inhibiting adult mollusks because it impedes both laying and spawning in addition to causing cercariae malformation and/or mortality. These findings led us to hypothesize that the substance can directly or indirectly act against all stages of the *S. mansoni* life cycle, indicating that BAR should be further explored in additional studies.

## Figures and Tables

**Table 1:** Percentage viability and inviability of snail embryos (F1) that survived treatment with the ether extract and BAR.

Substance	Concentration (µg/mL)	No. of Fecund Embryos *	Viability ^♦^ (%)	Inviability ^■^ (%)
Ctrl ^†^	–	379	0.5	99.5
Ctrl ^‡^	0.5%	300	1.5	98.5
	10.5	–	–	–
Ether extract	11	44	6.8	93.2
	12.5	26	0	100
	10	38	0	0
	11	–	–	–
BAR	11.5	–	–	–
	12	25	4	96
	12.5	–	–	–
	20	–	–	–

Legend: ^†^ Filtered and dechlorinated water; ^‡^ 0.5% ethanol + filtered and dechlorinated water; * number of embryos produced; **^♦^** hatched embryos; **^■^** malformation and/or mortality; – no spawning.

**Table 2:** Cercaricidal activity of BAR against *S. mansoni*.

Concentration (µg/mL)	15 min	30 min	60 min	120 min
**Ctrl**	−	−	−	−
**0.25**	−	+	+	+
**0.5**	−	+	++	++
**1**	−	++	+++	+++
**10**	++	+++	+++	+++
**100**	+++	+++	+++	+++
**N**	+++	+++	+++	+++

Legend: Complete elimination of cercariae (+++), elimination of more than 50% of cercariae (++), elimination of less than 50% of cercariae (+), and absence of lethality (−). Ctrl (0.5% ethanol + filtered and dechlorinated water), N (niclosamide).
